# Can Working Together Buffer Depression Among Older, Religious African American Couples?

**DOI:** 10.1093/geroni/igab046.3336

**Published:** 2021-12-17

**Authors:** Antonius Skipper, Andrew Rose, Ethan Jones, Alex Reeves, Jhazzmyn Joiner

**Affiliations:** 1 Georgia State University, Atlanta, Georgia, United States; 2 Texas Tech University, Lubbock, Texas, United States

## Abstract

Depression is a growing concern among older African Americans, as many within this group hesitate to seek professional help from psychiatrists or counselors. Instead, existing literature notes that older African Americans frequently utilize informal social support networks (e.g., church leaders) to respond to stress and buffer the negative effects of depression and depressive symptoms. Yet, little is known about the shared coping practices of older African American couples in relation to depression. Given the commonly noted high levels of religiosity among African Americans, this study examined communal coping as a mediator between sanctification and depression for older African American couples. This study utilized the dyadic data of 194 (146 married and 48 cohabiting) African American couples between the ages of 50 and 86 years. Capturing data with the Revised Sanctification of Marriage scale, the Communal Coping scale, and the Major Depression Inventory, bias-corrected bootstrap analysis revealed that men’s relationship sanctification and women’s depression was partially mediated by men’s, as well as the sum of men’s and women’s, communal coping in married couples. Further, men’s relationship sanctification and men’s depression was partially mediated by men’s, as well as the sum of men’s and women’s, communal coping. In addition, women’s sanctification was positively associated with men’s depression, directly. These findings are valuable in understanding the complex buffers, and contributors, to depression among older African American couples who may identify closely with religion but prefer the support of a partner over professional care.

